# Measuring the Degree of Overlap and Segregation among Multiple Probabilistic Home Ranges: A New Index with Illustrative Application to the Lesser Kestrel *Falco naumanni*

**DOI:** 10.3390/ani11102913

**Published:** 2021-10-09

**Authors:** Alessandro Ferrarini, Giuseppe Giglio, Stefania Caterina Pellegrino, Marco Gustin

**Affiliations:** Lipu—BirdLife Italy, Via Udine 3/a, I-43122 Parma, Italy; sgtpm@libero.it (A.F.); pinogiglio72@gmail.com (G.G.); stefaniacaterinapellegrino@gmail.com (S.C.P.)

**Keywords:** Alta Murgia, animal space use, biotelemetry, bivariate normal home range, Italy, multiple home range overlaps, overlap estimator, raptors, utilization distributions

## Abstract

**Simple Summary:**

Intraspecific and interspecific interactions regulate the extent and spatial patterns of animal home ranges. If we are able to estimate the home range overlap for a large number of individuals, populations and/or species, then we can readily identify important ecological properties, such as social network structure, competition during the breeding season, contact rates with implications for disease transmission, change in space use over time, interactions among different age classes and site fidelity for a particular individual, population or species. We can also evaluate the robustness of probabilistic home range assessment through the degree of overlap of several estimators. Accordingly, in this study, we first solve the issue of measuring the degree of overlap/segregation among a large number of probabilistic animal home ranges and provide a demonstrative case study.

**Abstract:**

Home range overlap/segregation has several important applications to wildlife conservation and management. In this work, we first address the issue of measuring the degree of overlap/segregation among an arbitrarily large number (i.e., *n* ≥ 2) of probabilistic animal home ranges (i.e., utilization distributions). This subject matter has recently been solved for home ranges measured as polygons (e.g., percent minimum convex polygons and multinuclear cores) but not yet for probabilistic ones. Accordingly, we introduce a novel index named the PGOI (probabilistic general overlap index), and its complement, the PGSI (probabilistic general segregation index), an index for computation of probabilistic home range overlap/segregation at individual, population and species levels. Whatever the number of probabilistic home ranges, the PGOI returns a single score ranging in the [0, 100] interval. We applied the PGOI to five lesser kestrels (*Falco naumanni*) at Santeramo in Colle (Apulia region; Southern Italy) as a case study. Our new index can be applied to any animal species and to home ranges derived from any type of probabilistic home range estimator.

## 1. Introduction

Animal space use has long been studied by ecologists [1,2] as it can influence intraspecific and interspecific interactions [3] and foraging efficiency [4]. Quantifying overlapping home ranges at individual, population and species levels is a key issue in studies on animal space use, as it provides a tool for testing hypotheses on territoriality [5], social network structure [6] and contact rates with implications for disease transmission [7].

There are several overlap indices in the scientific literature that return a matrix of pairwise overlaps between pairs of individuals, populations or species [8]. If home ranges are measured using polygons (e.g., minimum convex polygons and multinuclear cores), then the most common approach is percent overlap, i.e., the proportion of animal *i*’s home range that is overlapped by animal *j*’s home range [8]. Percent overlap is a pairwise measure, and, as such, when the number of individuals, populations or species is elevated, the resulting overlap matrix is overlarge and thus difficult to interpret. Recently, this issue has been solved through a general overlap index (*GOI* hereafter) for the computation of the overlap of multiple polygon home ranges [9]. Whatever the number of home ranges in the polygon format, the *GOI* always returns a single score ranging in the [0, 100] interval. However, home ranges can also be expressed in terms of the animal’s utilization distribution (*UD* hereafter), i.e., the probability density that an animal is found at a given grid cell or point within a certain space [10]. Overlap indices calculated using polygons do not take into account the individuals’ *UD*s, and thus they may result in biased estimates of overlap [8]. Several indices have been developed to measure home range overlap using *UD*s. Bhattacharyya’s affinity [11] is a statistical measure of affinity between two *UD*s, with values ranging from 0 (no overlap) to 1 (identical *UD*s). Further overlap indices that make use of *UD*s are the utilization distribution overlap index (*UDOI*) [12], the probability overlap index [13] and the volume of intersection index [14]. All these overlap indices present the desirable property in a range from 0 (no overlap) to 1 (identical *UD*s), with the exception of the *UDOI* that uses >1 if *UD*s are nonuniformly distributed and have a high degree of overlap. However, all these pairwise indices also share the same limit: they return an overlap matrix with pairwise overlaps between *UD*s rather than a single, easy-to-interpret score. 

To date, the scientific literature has not been able to propose a synthetic overlap index for probabilistic home ranges (*UD*s). In this work, we introduce, for the first time, a novel index named the *PGOI* (probabilistic general overlap index), and its complement to 100 (*PGSI,* probabilistic general segregation index), for the ready computation of overlap/segregation among an arbitrarily large number (i.e., *n* ≥ 2) of probabilistic animal home ranges at the individual, population and/or species levels. We applied the *PGOI* and *PGSI* to five lesser kestrels (*Falco naumanni*) as a case study, in order to estimate within-colony overlap/segregation in the urban colony of Santeramo in Colle (Apulia region; Southern Italy). This was a good case study as this colony belongs to the geographical area (Alta Murgia) with the most elevated density of lesser kestrels in urban areas worldwide [9]. The lesser kestrel is a small insectivorous raptor present among Annex I species of EU Wild Birds Directive 2009/147/EEC, which breeds in steppe-like grasslands and non-irrigated arable crops [15]. In Southern Italy, this raptor has been recently studied in the urban colonies of Gravina in Puglia, Altamura, Cassano delle Murge and Santeramo in Colle [16,17,18,19,20]. 

## 2. Materials and Methods

We tracked five birds at Santeramo in Colle between 13 and 29 June 2017 during the chick rearing period (Table 1). We fitted the birds with data loggers at their nest boxes. We used TechnoSmart GiPSy-4 and GiPSy-5 data loggers (23 mm × 15 mm × 6 mm, 5 g weight) to collect information about date, time, latitude, longitude, altitude and speed. Data acquisition occurred every three minutes following deployment. The weight of the loggers in relation to that of the tracked individuals was <4%. All devices were tied dorsally using a 2 mm large Teflon tape knotted with a triple simple knot. At the height of the sternum, two tapes were crossed without a knot so that the birds could fly freely. On no occasion did the application of data loggers have visible deleterious effects on the studied birds. In order to download the data from the data loggers, the birds were recaptured at their nest boxes after the batteries were exhausted.

We transferred GPS points into a GIS and estimated the individual probabilistic home ranges (*UD*s) using a bivariate normal home range model, which allowed for bivariate normal parameters to be estimated from autocorrelated location data [21] and thus accommodated the fact that telemetry data were autocorrelated.

In order to quantify probabilistic home range overlaps, we employed our probabilistic general overlap index (*PGOI*). The *PGOI* is a generalization of the general overlap index (*GOI*) [9] that allows for computation of overlap among an arbitrarily large number (*n* ≥ 2) of home ranges in polygon format. The *GOI* is calculated as

(1) $$GOI=100*\frac{Dist_{OBS}}{Dist_{MAX}}=100\times \frac{\sum_{i=1}^{n}A_{i}-\overset{n}{\underset{i=1}{\cup }}A_{i}}{\sum_{i=1}^{n}A_{i}-\max(A_{i})}$$

where *Dist_OBS_* and *Dist_MAX_* are the observed and maximum distances from the perfectly disjoint (i.e., non-overlapping) situation, respectively, $\sum A_{i}$ is the sum of home range extents, *n* is the number of home ranges, $\cup A_{i}$ corresponds to the union of the home range polygons, and *max*(*A_i_*) is the extent of the largest home range. Thus, the *GOI* measures the distance of the observed overlaps from a perfect overlap and a perfect non-overlap. If *Dist_OBS_* = 0 (i.e., perfect non-overlap), then *GOI* = 0; if *Dist_OBS_ = Dist_MAX_* (i.e., perfect overlap), then *GOI* = 100. In the intermediate cases, 0 < *GOI* < 100. A general segregation index (*GSI*) [9] can also be computed as the complement to 100 of the *GOI*:

(2) $$GSI=100-GOI=100\times (1-\frac{\sum_{i=1}^{n}A_{i}-\overset{n}{\underset{i=1}{\cup }}A_{i}}{\sum_{i=1}^{n}A_{i}-\max(A_{i})})$$

As both the *GOI* and *GSI* only consider the spatial domain of the individual home ranges and ignore the relative probabilities of use (*UDs*), in this study, we modified them to be applied to probabilistic home ranges. In probabilistic terms, in the case of perfect segregation, $\sum A_{i}$ becomes the sum of the *UD*s of all the home ranges under study. The sum of probabilities for the generic *UD_i_* is $\sum_{x}\sum_{y}UD_{i}\Delta x\Delta y$ (or $\left(\int_{x}\int_{y}UD_{i}dxdy\right)$ if Δ*x* ≅ 0 and Δ*y* ≅ 0) and is equal to 1 (or 100%) by definition; thus, the sum of the *UD*s of all the home ranges is simply equal to *n*, i.e., the number of probabilistic home ranges under study. Therefore, the term $\sum A_{i}$ is replaced by *n*. In the case of perfect overlap (i.e., identical *UD*s), *max*(*A_i_*) becomes $\max(\int_{x}\int_{y}UD_{i}dxdy)$, and because the sum of probabilities is equal to 1 for all the *UD*s, this is equal to 1. Thus, the term *max*(*A_i_*) is replaced by 1. In the intermediate case (i.e., partially overlapping *UD*s), $\cup A_{i}$ corresponds to the spatial union of the *UD*s, i.e., $\int_{x}\int_{y}\max(UD_{i})dxdy$, which is the probability surface where each grid cell assumes the maximum value among all the surfaces of the probabilistic home ranges. The *PGOI* is therefore calculated as

(3) $$PGOI=100*\frac{Dist_{OBS}}{Dist_{MAX}}=100\times \frac{n-\int_{x}\int_{y}\max(UD_{i})dxdy}{n-1}$$

As 1 ≤ $\int_{x}\int_{y}\max(UD_{i})dxdy$ ≤ *n*, the numerator is positive and also equal or less than the denominator. Therefore, the *PGOI* is forced to range from 0 (perfect home range segregation) to 100 (perfect overlap), while intermediate values indicate partially overlapping probabilistic home ranges. Finally, a probabilistic general segregation index (*PGSI*) can be computed as the complement to 100 of the *PGOI*:

(4) $$PGSI=100-PGOI=100\times (1-\frac{n-\int_{x}\int_{y}\max(UD_{i})dxdy}{n-1})$$

We applied the *PGOI* and *PGSI* to the probabilistic home ranges of the tracked raptors. In order to further elucidate the behavior of our overlap indices, we also simulated different overlap patterns by shifting the original home ranges, thus producing a broader range of spatial configurations for overlap analyses.

## 3. Results

**Figure 1:**
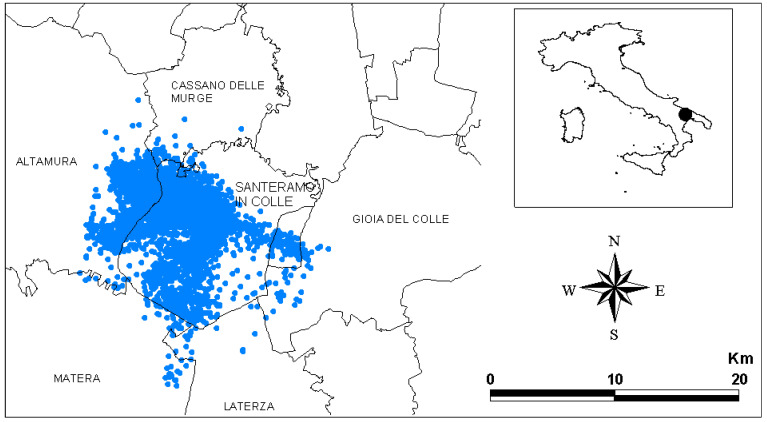
Study area (Santeramo in Colle, Apulia, Italy). Municipalities and GPS points (blue dots) of the tracked lesser kestrels belonging to the colony of Santeramo in Colle are shown.

In total, we collected 12,081 GPS points at Santeramo in Colle (Figure 1). The tracked lesser kestrels centered their activities within the municipality of Santeramo but also intruded into the neighboring municipalities (Altamura, Cassano delle Murge, Laterza, Gioia del Colle and Matera).

**Figure 2:**
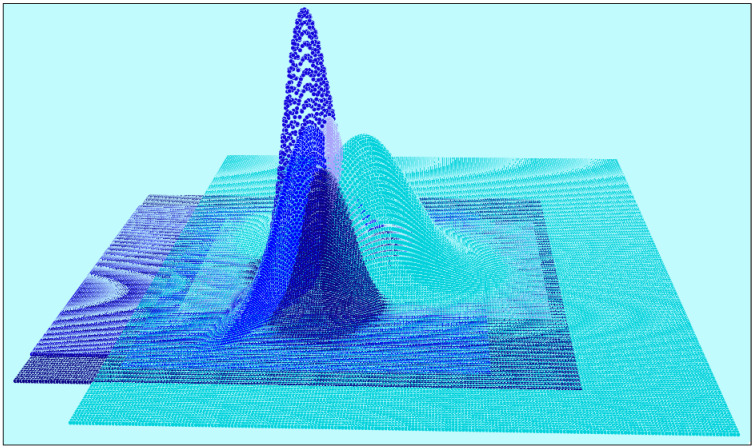
The five probability density functions (*UD*s) of the tracked lesser kestrels are shown in different shades of blue. The *X* and *Y* axes represent easting and northing, respectively, and the *Z* axis measures the probability density that a lesser kestrel is found at a given point in a space.

**Figure 3:**
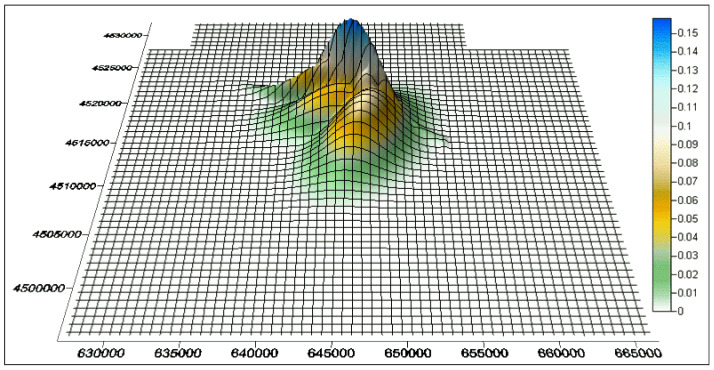
Union of the five *UD*s of the tracked lesser kestrels, i.e., the probability surface where each grid cell assumes the maximum value among all the surfaces of the probabilistic home ranges. On the *X* (easting) and *Y* (northing) axes, coordinates are expressed in meters. Probabilities are expressed in percentage.

The probabilistic home ranges of the tracked lesser kestrels were found to be highly overlapped (Figure 2). The union of the five *UD*s (i.e., the probability surface where each grid cell assumes the maximum value among all the surfaces of the probabilistic home ranges) is shown in Figure 3. The highest probability (0.0015 = 0.15%) was in correspondence with the lesser kestrels’ nests in the urban colony of Santeramo in Colle. The sum of probabilities of the union of the five *UD*s was 1.23936; thus, the *PGOI* was equal to 100 × (5 − 1.23936)/4 = 94.016%, and *PGSI* = 100% − 94.016% = 5.984%.

**Figure 4:**
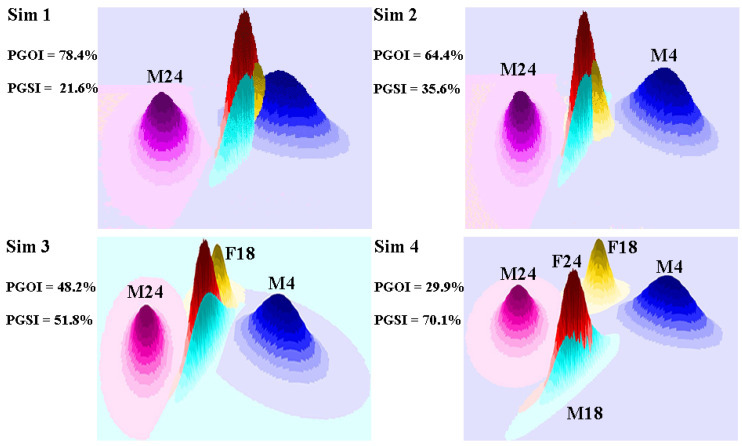
Resulting behavior of the *PGOI* and *PGSI* applied to simulated patterns of home range overlap. For each simulation, the IDs of the individuals whose probabilistic home ranges were shifted with respect to their original positions are shown. IDs are the same as those in Table 1.

We simulated four probabilistic home range patterns with a decreasing degree of overlap through the following rules: simulation (1), in which the home range of individual M24 was shifted 8 km north; simulation (2), where, in addition to M24, the home range of individual M4 was shifted 6 km south; simulation (3), where, in addition to M24 and M4, the home range of individual F18 was shifted 4 km east; simulation (4), where, in addition to M24, M4 and F18, the home ranges of individuals M18 and F24 were shifted 9 and 10 km west, respectively. The simulated home range patterns depict the behavior of the *PGOI* and *PGSI* for different levels of probabilistic overlap (Figure 4). The *PGOI* ranged from 29.9% (simulation 4) to 78.4% (simulation 1).

## 4. Discussion

Home range overlap is of great importance in ecological studies. In fact, it has been used to evaluate habitat quality [22], social associations relative to kinship [23], mechanisms of predation [24], coexistence [25] and competition [26]. The *UD* provides a useful summary of space use for a given individual and thus plays a key role when measuring the degree of space use sharing among individuals, populations and/or species, or the degree of site fidelity across years or seasons [14]. However, the issue of estimating the degree of overlap among multiple probabilistic home ranges has remained unsolved to date. Accordingly, we first introduced a non-pairwise metric of overlap/segregation among multiple *UD*s and applied it to the lesser kestrel colony of Santeramo in Colle as a case study. 

In our previous study [9], we found that the overlap among the minimum convex polygons of the lesser kestrels at Santeramo in Colle was 81.38%. In this study, using probabilistic home ranges, we found that the overlap figure stood at 94.016%. In both cases, the degree of overlap among the individuals of this colony was very elevated; however, estimation based on *UD*s is closer to reality because it takes into account the relative frequency of use across the landscape.

The *PGOI* and *PGSI* generalize previous overlap/segregation indices, namely, the *GOI* and *GSI* [9], which can be readily applied to probabilistic home ranges as well. As for the *GOI* and *GSI*, the rationale behind these two indices is simple: given *n* probabilistic home ranges, the *PGOI* measures the distance of the observed overlaps from two spatial configurations, perfect segregation and perfect overlap, and always returns a single overlap measure. 

Several authors [12,27] argued that any overlap index should produce measures consistent with one’s intuition of overlap and also be easy to interpret. Accordingly, the ecological interpretation of just one overlap index is much easier if compared to an *n* × *n* pairwise overlap matrix computed through standard pairwise overlap indices for probabilistic ranges. We also argue that one overlap index is more effective if estimates of the overlap are to be meaningfully compared across several studies. In addition, the *PGOI* and *PGSI* are also computationally fast as they just require calculating the union of the *UD*s within a GIS (Figure 3). 

The *PGOI* corresponds to the linear equation *Y* = 100 × (*n* − X)/(*n* − 1), where 1 ≤ *X* ≤ *n*, and 0 ≤ *Y* ≤ 100. The first derivative of the *PGOI* with respect to the spatial union of the *UD*s (i.e., $\cup A_{i}$) is thus equal to −100/(*n* − 1); therefore, every unitary increase or decrease in $\cup A_{i}$ (due, for instance, to the addition of further *UDs* or changes to some *UDs* over time) determines a decrease/increase in the *PGOI* that is slower when the number of home ranges (*n*) is elevated, and vice versa. The rationale is simple: As the number of *UDs* increases, the estimation of the probabilistic overlap at the individual, population or species level is more robust, and thus the *PGOI* (and the *PGSI* as well) becomes less sensitive to changes in some *UDs*. Instead, the *PGOI* (and the *PGSI* too) becomes more prone to being modified by updates to the *UDs* when the estimation of the probabilistic overlap is less robust due to the limited number of home ranges under study. 

We estimated the probabilistic home ranges using a bivariate normal home range model, although the *PGOI* and *PGSI* can be applied to *UD*s derived from any type of probabilistic home range estimator, e.g., Brownian bridge movement models [28,29] and kernel density estimation [30,31]. Although the *PGOI* and *PGSI* are complementary, the *PGOI* seems more appropriate for application to species with elevated intraspecific overlaps, as in the case of central place foragers such as lesser kestrels; in the case of species with low overlaps, the *PGSI* is more suitable to assess the degree of probabilistic home range segregation. We applied the *PGOI* and *PGSI* to raptors, but they can be applied to probabilistic home ranges of any animal species. 

## 5. Conclusions

The proposed overlap index first solves the question of generalizing pairwise measures of probabilistic home range overlap to a single measure of overlap. The *PGOI* and *PGSI* thus represent a tool for easy ecological interpretation of overlap/segregation among multiple probabilistic home ranges, which is especially useful when the number of *UD*s is elevated. Both the *PGOI* and *PGSI* can be utilized not only to measure the degree of overlap among *n* different individuals (species or populations) but also to plainly quantify how the individuals’ home range overlap changes over time, e.g., between life history stages, or before and after experimental manipulations. Another important application of our new index might be the evaluation of the robustness of home range assessment through the degree of overlap of several probabilistic estimators. 

## Figures and Tables

**Table 1:** Description of the tracked lesser kestrels.

ID	Sex	Weight (g)	Start Date of Tracking	End Date of Tracking	No. of GPS Points
M4	M	124	16 June 2017	22 June 2017	2765
F18	F	155	13 June 2017	16 June 2017	1375
M18	M	135	13 June 2017	16 June 2017	1417
F24	F	120	22 June 2017	29 June 2017	3311
M24	M	116	22 June 2017	29 June 2017	3213

## Data Availability

The dataset used in this study is available from the first author on reasonable request.
